# GLUT-1 overexpression as an unfavorable prognostic biomarker in patients with colorectal cancer

**DOI:** 10.18632/oncotarget.14352

**Published:** 2016-12-29

**Authors:** Jing Yang, Jing Wen, Tian Tian, Zhongsheng Lu, Yao Wang, Zikai Wang, Xiangdong Wang, Yunsheng Yang

**Affiliations:** ^1^ Department of Gastroenterology and Hepatology, Chinese PLA General Hospital, Beijing, China; ^2^ Department of Gastroenterology and Hepatology, Chinese PLA 261 Hospital, Beijing, China; ^3^ Nanlou Department of Respiratory Disease, Chinese PLA General Hospital, Beijing, China; ^4^ Department of Immunology/Bio-therapeutic, Institute of Basic Medicine, Chinese PLA General Hospital, Beijing, China

**Keywords:** meta-analysis, biomarker, colorectal cancer, survival, GLUT-1

## Abstract

**Background:**

Glucose transporter-1 (GLUT-1) exhibits altered expression in colorectal cancer (CRC). The aim of this study was to explore the association between GLUT-1 and survival conditions, as well as clinical features in CRC by meta-analysis.

**Materials and Methods:**

Relevant studies were searched through predefined strategies, hazard ratios (HRs), odds ratios (ORs), and their 95% confidence intervals (CIs) were used as effective measures.

**Results:**

A total of 14 studies with 2,077 patients were included in this meta-analysis. The results showed that GLUT-1 was not significantly associated with overall survival (OS) (HR=1.28, 95% CI=0.86–1.91, p=0.22) or disease-free survival (DFS) (HR=1.71, 95% CI=0.78–3.72, p=0.179). However, subgroup analysis indicated that GLUT-1 was a significant biomarker for poor DFS in rectal cancer (HR=2.47, 95% CI=1.21–5.05, p=0.013). GLUT-1 expression was also found to be significantly correlated with the presence of lymph node metastasis (n=8, OR=2.14, 95% CI=1.66–2.75, p<0.001), T stage (n=6, OR=1.73, 95% CI=1.17–2.58, p=0.007), higher Dukes stage (n=5, OR=2.92, 95% CI=2.16–3.95, p<0.001), female sex (n=4, OR=2.92, 95% CI=2.16–3.95, p<0.001), and presence of liver metastasis (n=3, OR=1.82, 95% CI=1.06–3.12, p=0.03).

**Conclusion:**

In conclusion, this meta-analysis showed that GLUT-1 was associated with poor DFS in rectal cancer (RC). Furthermore, GLUT-1 was also an indicator of aggressive clinical features in CRC.

## INTRODUCTION

Colorectal cancer (CRC) is the third most prevalent cancer and the second leading cause of cancer-related death worldwide [1]. It is estimated that 1.36 million new cases and 693,900 deaths occurred in 2012 [2]. Although significant advances have been achieved in the treatment of CRC, the 5-year survival rate is 64% [3, 4]. Confounding this is that fact that about one-fifth of CRC patients are in metastatic disease at first diagnosis. Further clarification of the biological mechanisms of cancer progression could help to identify effective biomarkers for prognostication.

Malignant cells often have elevated metabolic rates than normal cells [5], and glucose is known to serve as a substrate and a regulator of metabolic pathways in cellular metabolism [6]. Glucose transporters are membrane transporter proteins that catalyze the facilitative bidirectional transfer of their substrates across membranes [7]. Glucose transporter-1 (GLUT-1) is the first identified member of glucose transporter family, and the most intensively studied of all membrane transport proteins [8]; it was reported to be overexpressed in various malignancies [9–11]. Previous studies [12–15] also showed the prognostic value of GLUT-1 expression in CRC; however, there was little consistency in the results that were presented. Limited sample sizes or other inconsistent study parameters could be the potential factors leading to these discrepant findings. Therefore, we collected all relevant studies and performed a quantitative meta-analysis to investigate the prognostic and clinical role of GLUT-1 in CRC. To our knowledge, this meta-analysis is the first one on this subject to date.

## RESULTS

### Studies selection and characteristics

**Figure 1:**
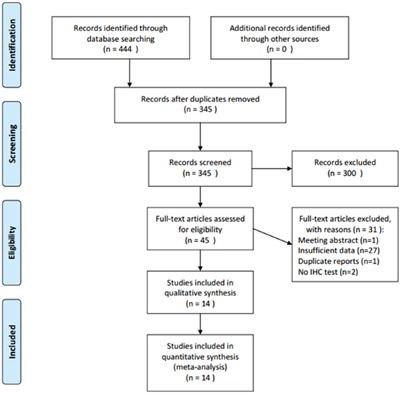
Flow diagram for articles included in this meta-analysis

Four hundred and forty-four records were identified through initial searching as described in the methods. After duplicate records were discarded, 345 records were left for screening, of which 300 records were then removed by title and/or abstract inspection. Forty-five records were further evaluated by full-text reading, and 31 studies were excluded either owing to being a meeting abstract (n=1), lacking necessary information (n=27), being duplicate studies (n=1), or not using immunohistochemistry (IHC) methods (n=2). Finally, 14 studies [12–25] published from 1998 to 2016 were included in the meta-analysis. The article selection process was shown in Figure 1. The included studies were from 10 countries with a total sample size of 2,077, and all studies used IHC to detect GLUT-1. Nine studies [12–14, 16, 18–20, 22, 25] recruited patients with CRC and 5 studies [15, 17, 21, 23, 24] were performed on patients with rectal cancer (RC). Newcastle-Ottawa Scale (NOS) scores ranged from 7 to 9, indicating that all included studies were high quality studies. The baseline characteristics of included studies were demonstrated in Table 1.

**Table 1:** Basic information of included studies

Study	Year	Country	No. of patients	Sex (M/F)	Cancer type	Method	Treatment	NOS score	GLUT-1 (+) threshold
Haber	1998	USA	112	60/52	CRC	IHC	Surgical resection	7	Immunostaining >50%
Furudoi	2001	Japan	152	94/58	CRC	IHC	Surgical resection	8	Immunostaining >47.9%
Cooper	2003	Turkey	43	29/14	RC	IHC	Surgical resection	7	Immunostaining >0%
Yuan	2003	China	42	24/18	CRC	IHC	Surgical resection	7	Immunostaining >50%
Zhou	2005	China	60	32/28	CRC	IHC	Surgical resection	9	Immunostaining >10%
Cleven	2007	The Netherlands	133	55/78	CRC	IHC	Surgical resection	8	Immunostaining >5%
Wincewicz	2007	Poland	123	65/58	CRC	IHC	Surgical resection	8	Immunostaining >10%
Havelund	2011	Denmark	86	49/37	RC	IHC	Chemoradiotherapy	7	Immunostaining >50%
Jun	2011	Korea	515	293/222	CRC	IHC	Surgical resection	8	Immunostaining >50%
Korkeila	2011	Finland	178	104/74	RC	IHC	Surgical resection	8	Immunostaining >10%
Hong	2012	Korea	44	24/20	RC	IHC	Surgical resection	7	Immunostaining >10%
Lastraioli	2012	Italy	135	63/72	CRC	IHC	Surgical resection	7	Immunostaining >50%
Shim	2013	Korea	104	71/33	RC	IHC	Chemoradiotherapy	7	Immunostaining >50%
Goos	2016	The Netherlands	350	NR	CRC	IHC	Surgical resection	8	Immunostaining >50%

Abbreviations: CRC, colorectal cancer; RC, rectal cancer; IHC: immunohistochemical staining; NOS, Newcastle-Ottawa Scale.

### GLUT-1 and overall survival

**Figure 2:**
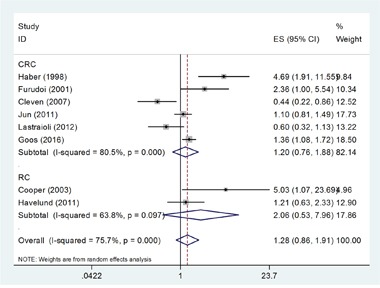
Forest plot diagrams of hazard ratios for correlations between GLUT-1 expression and OS The forest plot was stratified by cancer type. The pooled HR and 95%CI for OS were on the overall horizontal line. The horizontal axis indicates the pooled HR. ES (effect size) is HR in this figure.

Eight studies [13, 14, 16, 17, 19, 21, 22, 25] investigated the correlation of GLUT-1 expression and overall survival (OS) in a total of 1,526 patients. Owing to significant heterogeneity (*I*^2^=75.7%, P_h_<0.001; Table 2), a random-effects model was used. The results showed that there was no significant correlation between GLUT-1 expression and OS in CRC (Hazard ratio [HR] =1.28, 95% confidence interval [CI] = 0.86–1.91, p=0.22; Table 2, Figure 2). To further investigate the connection of GLUT-1 and OS, we conducted subgroup analyses. RC is a subtype of CRC and subgroup analysis on cancer types of CRC and RC was performed to clarify the specific role of GLUT-1 in RC. The pooled data demonstrated that GLUT-1 still had no significant association with OS irrespective of location, cancer type, and treatment (Table 2).

**Table 2:** Main results of meta-analysis for colorectal cancer

Variables	No. of studies	Effects model	HR (95%CI)	p	Heterogeneity	
					*I*^2^(%)	P_h_
OS	8	Random	1.28(0.86-1.91)	0.22	75.7	<0.001
Location						
Western countries	5	Random	1.11(0.61-2.03)	0.734	82.5	<0.001
Eastern countries	3	Random	1.86(0.83-4.17)	0.132	66.1	0.053
Cancer type						
CRC	6	Random	1.2(0.76-1.88)	0.44	80.5	<0.001
RC	2	Random	2.06(0.53-7.96)	0.294	63.8	0.097
Treatment						
Surgical resection	7	Random	1.31(0.83-2.06)	0.244	79.2	<0.001
Chemoradiotherapy	1	-	1.21(0.63-2.33)	0.568	-	-
DFS	4	Random	1.71(0.78-3.72)	0.179	61.8	0.049
Location						
Western countries	1	-	0.87(0.24-3.12)	0.831	-	-
Eastern countries	3	Random	2.16(0.75-6.18)	0.152	73.5	0.023
Cancer type						
CRC	1	-	1.12(0.86-1.46)	0.414	-	-
RC	3	Fixed	2.47(1.21-5.05)	0.013	46.4	0.155
Treatment						
Surgical resection	3	Fixed	1.13(0.87-1.46)	0.364	0	0.476
Chemoradiotherapy	1		4.01(1.55-10.37)	0.004	-	-

### GLUT-1 and disease-free survival

**Figure 3:**
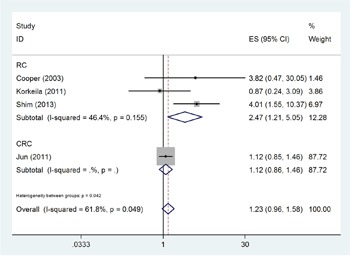
Forest plot diagrams of hazard ratios for correlations between GLUT-1 expression and DFS The forest plot was stratified by cancer type. The pooled HR and 95%CI for DFS were on the overall horizontal line. The horizontal axis indicates the pooled HR. ES (effect size) is HR in this figure.

There were 4 studies [17, 22–24] with 840 subjects reporting the correlation between GLUT-1 expression and disease-free survival (DFS). Because of heterogeneity (*I*^2^=61.8%, P_h_=0.049; Table 2), random-effects model was used for this calculation. The pooled HR was 1.71, with 95% CI=0.78–3.72, p=0.179 (Table 2, Figure 3). Subgroup analyses were also performed, the results of which showed that GLUT-1 was significantly associated with shorter DFS in rectal cancer (HR=2.47, 95% CI=1.21–5.05, p=0.013, Table 2, Figure 3). However, no significant association was found when stratified by location and treatment.

### GLUT-1 and clinical features

**Figure 4:**
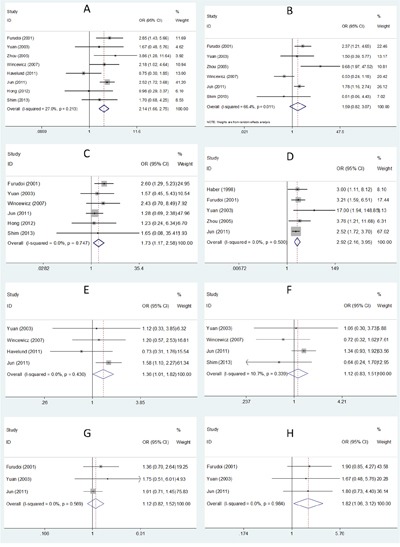
Association between GLUT-1 expression and clinicopathological parameters in CRC **A**. lymph node metastasis; **B**. differentiation; **C**. T stage; **D**. Dukes stage; **E**. sex; **F**. age; **G**. tumor size; **H**. liver metastasis.

Nine studies [12, 13, 15, 16, 18, 20–22, 24] reported the association between GLUT-1 and clinical features. A total of 8 clinical features were investigated, which were age (≥60 vs. <60), sex (female vs. male), lymph node metastasis (yes vs. no), differentiation (poor vs. moderate/well), T stage (T3+T4 vs. T1+T2), Dukes stage (C+D vs. A+B), tumor size (≥5cm vs. <5cm), and liver metastasis (yes vs. no). The results showed that GLUT-1 expression was significantly correlated with presence of lymph node metastasis (n=8, OR=2.14, 95% CI=1.66–2.75, p<0.001, Figure 4), T stage (n=6, OR=1.73, 95% CI=1.17–2.58, p=0.007, Figure 4), higher Dukes stage (n=5, OR=2.92, 95% CI=2.16–3.95, p<0.001, Figure 4), female sex (n=4, OR=2.92, 95% CI=2.16–3.95, p<0.001, Figure 4), and presence of liver metastasis (n=3, OR=1.82, 95% CI=1.06–3.12, p=0.03, Figure 4). However, there was no significant connection between GLUT-1 and other clinical features including tumor differentiation (n=6, OR=1.59, 95% CI=0.82–3.07, p=0.166, Figure 4), age (n=4, OR=1.12, 95% CI=0.83–1.51, p=0.455, Figure 4), or tumor size (n=3, OR=1.12, 95% CI=0.82–1.52, p=0.48, Figure 4).

### Publication bias

**Figure 5:**
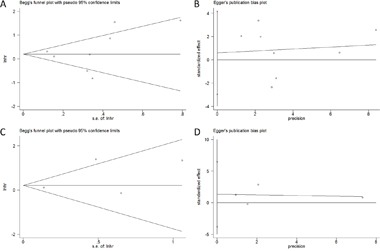
Publication bias in the meta-analysis **A**. Begg's test for OS; **B**. Egger's test for OS; **C**. Begg's test for DFS; **D**. Egger's test for DFS.

Begg's test [26] and Egger's test [27] were used to detect potential publication bias. For OS, the p values for Begg's test and Egger's test were 0.266 and 0.699, respectively (Figure 5). For DFS, Begg's p was 0.734 and Egger's p was 0.382, respectively (Figure 5). The results demonstrated that there was no evidence of significant bias in this meta-analysis, and therefore, our results were statistically reliable.

## DISCUSSION

To our knowledge, the current meta-analysis was the first to investigate the prognostic significance of GLUT-1 expression in CRC. Our study demonstrated that GLUT-1 was associated with poor DFS in rectal cancer, but had a non-significant correlation with OS. Furthermore, GLUT-1 overexpression was positively connected with lymph node metastasis, T stage, higher Dukes stage, female sex, and presence of liver metastasis. Taken together, these results indicated that GLUT-1 was a promising prognostic biomarker for shorter DFS and aggressive clinical parameters.

GLUT-1 mediates basal glucose transport in cancer cells and provides glucose for energy metabolism [28]. Inducing angiogenesis is a hallmark of cancer and is often correlated to cancer progression [5]. When cancer cells outgrow the existing vasculature, then hypoxia occurs, and as a result of this, cancer cells respond to hypoxic conditions through activating genes that are responsible for glucose transport [29]. Malignant cells require high energy levels to proliferate, and GLUT-1 was found to be overexpressed in various cancer types including prostate cancer, gastric cancer, breast cancer, head and neck cancer, and lung cancer [30]. Previous studies also showed that GLUT-1 overexpression was an indicator of poor prognosis in different cancers [11, 31–34]. The present meta-analysis identified GLUT-1 as a significant biomarker for DFS in rectal cancer. Our results were in accordance with previous findings in other cancer types, and owing to the fact that no meta-analysis on GLUT-1 and prognosis of patients with cancer has been reported, we could not compare our results with other similar meta-analyses. We also found GLUT-1 was correlated with a variety of clinical parameters, which suggest that GLUT-1 could be a potential marker for aggressive biological behavior of cancer cells.

Several limitations still need to be acknowledged. First, significant heterogeneity among studies was detected on OS and DFS analyses. Although we selected eligible studies using uniform criteria, inherent differences among studies still existed. Second, the sample size was relatively small, where only four studies were included when the correlation between GLUT-1 and DFS was analyzed. Although Begg's test and Egger's test suggested non-significant publication bias, selection bias could be possibly exist owing to limited sample size.

In conclusion, this meta-analysis showed that GLUT-1 was associated with poor DFS in rectal cancer. In addition, GLUT-1 was also an indicator of aggressive clinical features in CRC. Because several limitations exist in this study, further investigations are required to warrant our findings.

## MATERIALS AND METHODS

### Search strategy and study selection

This meta-analysis was carried out in line with the Preferred Reporting Items for Systematic Reviews and Meta-Analyses (PRISMA) statement [35]. Electronic platforms of PubMed, Embase, Web of Science, and China National Knowledge Infrastructure (CNKI) were thoroughly searched to September 8, 2016. The search items were as follows: “Glucose transporter-1”, “GLUT-1”, “SLC2A1”, “colorectal cancer”, “colon cancer”, “rectal cancer”, and “;colorectal neoplasms”[MeSH Terms]. The reference lists of all retrieved articles were screened to identify other relevant studies. The inclusion criteria in the meta-analysis were as follows: (1) studies investigating the association between GLUT-1 expression and survival outcomes or clinical features; (2) studies where the diagnosis of CRC was confirmed via pathology reports; (3) studies that reported patients with either colorectal cancer or colon cancer or rectal cancer; (4) studies where the hazard ratios (HRs) and 95% confidence intervals (CIs) for survival analysis were directly reported or could be calculated using Parmar's methods [36]; (5) studies measuring GLUT-1 expression via immunohistochemistry (IHC); and (6) studies published as full-text articles in either English or Chinese. Exclusion criteria were: (1) reviews, case reports, letters, and meeting abstracts; (2) studies not using IHC to detect GLUT-1; (3) studies lacking necessary data for calculation.

### Data extraction and quality assessment

Two independent investigators extracted the following information from eligible studies: first author's name, year of publication, study country, sample size, sex, cancer type, detection method, treatment methods, and research period. Discrepancies between the two investigators were resolved by discussion. The qualities of included studies were evaluated by using NOS [37]. The NOS assessed a study on three aspects: selection (4 stars), comparability (2 stars), and outcome (3 stars). The maximal score is 9 stars and studies with ≥7 stars were assigned as high quality studies.

### Statistical analysis

All statistical analyses were performed using Stata 12.0 (Stata Corporation, College Station, TX, USA). HRs and 95% CIs were used to evaluate the effect of GLUT-1 expression on overall survival (OS) and disease-free survival (DFS) in CRC patients. HR >1 without 95% CI overlapping 1 indicates a significant association between high GLUT-1 expression and poor outcomes. Heterogeneity among studies was tested using Cochran Q and *I*^2^ statistics. If p-value for heterogeneity (P_h_) <0.10 and *I*^2^>50% indicate significant heterogeneity, then a random-effects model was used; otherwise, a fixed-effects model was utilized. For further analysis, subgroup analyses according to location, cancer type, and treatment were conducted. Odds ratios (ORs) and 95% CIs were used to calculated the relevance of GLUT-1 and clinical features. Publication bias was tested by using Begg's funnel plot test [26] and Egger's test [27]. P<0.05 was considered as statistically significant.
